# Identification of microorganisms by a rapid PCR panel from positive blood cultures leads to faster optimal antimicrobial therapy – a before-after study

**DOI:** 10.1186/s12879-023-08732-9

**Published:** 2023-10-26

**Authors:** Jessica Agnetti, Andrea C. Büchler, Michael Osthoff, Fabrice Helfenstein, Maja Weisser, Martin Siegemund, Stefano Bassetti, Roland Bingisser, Dirk J. Schaefer, Martin Clauss, Vladimira Hinic, Sarah Tschudin-Sutter, Veronika Bättig, Nina Khanna, Adrian Egli

**Affiliations:** 1 Clinical Bacteriology and Mycology, University Hospital Basel, Basel, Switzerland; 2 Department of Biomedicine, Applied Microbiology Research, University of Basel, Basel, Switzerland; 3 Infectious Diseases and Hospital Epidemiology, University of Basel and University Hospital Basel, Basel, Switzerland; 4 Internal Medicine, University of Basel and University Hospital Basel, Basel, Switzerland; 5 Department of Clinical Research, University of Basel and University Hospital Basel, Basel, Switzerland; 6 Intensive Care Medicine, University Hospital Basel, Basel, Switzerland; 7 Emergency Medicine, University Hospital Basel, Basel, Switzerland; 8 Plastic, Reconstructive, Aesthetic Surgery and Hand Surgery, University Hospital Basel, Basel, Switzerland; 9 Center for Musculoskeletal Infections (ZMSI), University Hospital Basel, Basel, Switzerland; 10 Orthopaedics and Traumatology, University Hospital Basel, Basel, Switzerland; 11 Present Address: Institute for Medical Microbiology, University of Zurich, Gloriastrasse 28/30, CH-8006 Zurich, Switzerland

**Keywords:** BioFire FilmArray, Blood culture, Sepsis, Antimicrobial stewardship, Anti-infective agents, PCR panel

## Abstract

**Background:**

The BioFire® FilmArray® Blood Culture Identification Panel 1 (BF-FA-BCIP) detects microorganisms with high accuracy in positive blood cultures (BC) – a key step in the management of patients with suspected bacteraemia. We aimed to compare the time to optimal antimicrobial therapy (OAT) for the BF-FA-BCIP vs. standard culture-based identification.

**Methods:**

In this retrospective single-centre study with a before-after design, 386 positive BC cases with identification by BF-FA-BCIP were compared to 414 controls with culture-based identification. The primary endpoint was the time from BC sampling to OAT. Secondary endpoints were time to effective therapy, length of stay, (re-)admission to ICU, in-hospital and 30-day mortality. Outcomes were assessed using Cox proportional hazard models and logistic regressions.

**Results:**

Baseline characteristics of included adult inpatients were comparable. Main sources of bacteraemia were urinary tract and intra-abdominal infection (19.2% vs. 22.0% and 16.8% vs. 15.7%, for cases and controls, respectively). Median (95%CI) time to OAT was 25.5 (21.0–31.2) hours with BF-FA-BCIP compared to 45.7 (37.7–51.4) hours with culture-based identification. We observed no significant difference for secondary outcomes.

**Conclusions:**

Rapid microorganism identification by BF-FA-BCIP was associated with a median 20-h earlier initiation of OAT in patients with positive BC. No impact on length of stay and mortality was noted.

**Trial registration:**

Clinicaltrials.gov, NCT04156633, registered on November 5, 2019.

**Supplementary Information:**

The online version contains supplementary material available at 10.1186/s12879-023-08732-9.

## Background

Estimated among the top seven causes of death in Europe and North America, bloodstream infections (BSI) continue to represent a major healthcare challenge and are expected to be an increasing concern owing to rising incidence rates in an aging population [1]. Suspicion of serious infection accompanied by sepsis and septic shock often requires the use of broad-spectrum antimicrobials [2, 3]. The identification of microorganisms in positive blood cultures (BC) remains a key diagnostic step both for ensuring effective therapy vital for patient outcome, as well as allowing de-escalation or discontinuation of unnecessary but potentially toxic therapy [4]. Antimicrobial stewardship plays a central role in optimising patient management and in preventing the emergence of multi-drug resistant organisms (MDRO) through the responsible use of antimicrobials [5]. One of the main limiting factors for timely interventions is, however, the time to microorganism identification.

Although culture-based techniques remain the gold-standard, polymerase chain reaction (PCR)-based multiplex panels are increasingly used complimentarily allowing detection of microorganisms with high accuracy within few hours from BC positivity [6]. Several studies show evidence for a beneficial impact of bioMérieux’s BioFire FilmArray blood culture identification panel 1 (BF-FA-BCIP) on the time to optimal antimicrobial therapy [7–10]. A prospective randomised trial demonstrated the importance of a combined implementation with an antibiotic stewardship intervention for an enhanced effect [11]. An impediment to widespread application of such methods are the substantial costs [12]. Outcome studies remain crucial to establish best practices and prevent medical overuse.

The present study evaluates the clinical impact of the BF-FA-BCIP in its function as an integral part of the antimicrobial stewardship program (ASP) at a tertiary care centre in a setting with low antimicrobial resistance. We aimed to measure the impact on the management of hospitalised patients with a positive BC by comparing the time to optimal antimicrobial therapy (OAT) with culture-based identification.

## Methods

This retrospective before-after study was performed at the University Hospital of Basel, a 755-bed tertiary care centre with approximately 35,000 admissions per year. The study protocol was approved by the Ethics Committee of Northwest and Central Switzerland (Ethikkommission Nordwest- und Zentralschweiz Project-ID 2019–01860) with a waiver of informed consent. The study was registered at clinicaltrials.gov (NCT04156633).

In 2018, the BF-FA-BCIP was introduced alongside conventional culture-based identification of positive BC. Balancing costs against benefits, the BF-FA-BCIP was implemented during hours of less staff availability (Supplementary Fig. 1). Over the observation period of each one year, patients with a positive BC before (08/2017 to 07/2018) were compared to patients after BF-FA-BCIP implementation (11/2018 to 10/2019). The time gap between the two periods reflects the stepwise introduction of the BF-FA-BCIP.

All hospitalised patients aged > 18 years with a positive BC were eligible for inclusion in the study. To ensure comparability, the time of observation was limited to the hours of BF-FA-BCIP implementation during both before and after study periods (Suppl. Figure 1). Blood cultures flagging positive outside of these hours were processed by a different protocol which has been described elsewhere [13]. Only a patient’s first positive BC was considered, unless there was more than one week between the episodes, then the positive BC was counted as a new event and the patient qualified again. Patients first admitted to other hospitals, patients who died before BC positivity and patients with documented refusal of the general consent were excluded. According to the hospital’s internal standard of procedure for the BF-FA-BCIP, BC with a time to positivity of more than 36 h were excluded, except for fungal pathogens.

The primary outcome was the time in hours to implementation of the OAT. Secondary endpoints were the time to effective therapy, length of hospital stay, (re-)admission to intensive or intermediate care unit (ICU/IMC), all-cause in-hospital and 30-day mortality. Primary and secondary endpoints were calculated from the time of BC collection. Outcomes were also analysed in subgroups based on hospital wards, patients with neutropenia, patients with a contaminated BC and identified microorganisms. Optimal therapy was defined as the most narrow-spectrum antimicrobial for the identified microorganism(s), considering patient characteristics (e.g., allergies, neutropenia), susceptibility results and local resistance patterns (Suppl. Table 1). In case of contaminated BC, optimal therapy was defined as the discontinuation of unnecessary antimicrobial therapy. An infectious disease specialist prospectively evaluated the clinical significance of all positive BC and determined if the detected microorganism corresponded to a true bacteraemia or contamination based on microbiological information, e.g., number of positive BC sets and identification of a common commensal microorganism [14, 15]. Effective therapy was defined as active antimicrobial therapy according to in vitro susceptibility testing following EUCAST [16, 17] and CLSI [18, 19] recommendations.

Data on patient characteristics (including the quick sequential organ failure assessment (qSOFA) score [20] and the Charlson comorbidity index (CCI) [21]), as well as microbiological data and information on antimicrobial therapy was extracted retrospectively from the electronic health records. To ensure a consistent evaluation of antimicrobial therapy, antimicrobials were pre-classified by a clinical review board consisting of three infectious disease specialists into broad, narrow, and optimal according to the pathogen identified (Suppl. Table 1). The review board evaluated the antimicrobial therapy in the case of rare microorganisms, MDRO (defined as in vitro resistance to at least one agent in three or more antimicrobial categories [22]), type I and IV allergies, and fever in patients with severe neutropenia (absolute neutrophil count of < 500/µl) or acute graft-versus-host disease and proceeded by consensus decision.

The BF-FA-BCIP was implemented in close cooperation with the antimicrobial stewardship programme (ASP) at our centre and integrated into daily ASP routine. Infectious disease specialists provide feedback on antimicrobial therapy for all positive BC during daytime (11 am to 6 pm) from Monday through Sunday. During the ‘before’ study period, the treating physician was contacted by the infectious disease specialist by phone and in special cases followed up by a written consultation note. Since the implementation of the BF-FA-BCIP, a written consultation note was provided consistently after oral communication.

### Microbiological analysis

BC bottles were incubated using the automated BACT/ALERT® VIRTUO® system (BactAlert FA/FN plus, bioMérieux, Marcy l’Etoile, France). Following positive signalling, BC were submitted to Gram stain and microscopy. In the intervention group, the BioFire® FilmArray® Blood Culture Identification Panel 1 (bioMérieux Marcy l’Etoile, France) was performed according to the manufacturer’s instructions. In both groups (cases and controls), samples were cultured overnight with subsequent identification of the microorganisms by Matrix Assisted Laser Desorption Ionisation—Time of Flight mass spectrometry (MALDI-TOF MS) using the Microflex System (Bruker, Bremen, Germany). In positive BC with Gram-negative rods, species identification was based on biochemical profiling (VITEK2 GN card, bioMérieux). For susceptibility testing the VITEK2 AST-N242, -P586 and -P636 cards and MIC Test Strips (Liofilchem, Italy) were used. Further details are described in Supplementary material 1.1.

### Statistical analysis

All analyses were defined a priori in a statistical analysis plan written by FH and reviewed by an independent statistician according to the standard operating procedures at the DKF Basel. In all multivariable models independent variables were pre-selected based on clinical relevance. Time to OAT, time to effective therapy and length of stay were assessed using a cause-specific Cox proportional hazard model including the diagnostic method and the following independent variables: hospital ward (ICU/IMC, surgical, medical), neutropenia, previous MDRO colonisation, and the CCI (for definitions, see above). A competing risk model according to Fine & Gray [23], whereby missing values were censored at the date of death or discharge (i.e. competing events), yielded qualitatively similar results. Binary outcomes were assessed using a logistic model. Sensitivity and specificity of the BF-FA-BCIP were assessed according to Blaker [24]. Estimates are presented along with their 95% confidence intervals (CI) and *p*-values are two-sided.

## Results

From November 2018 to October 2019 the BF-FA-BCIP was performed on 490 positive BC within the observed hours (Suppl. Figure 1). 386 (78.8%) positive BC episodes met inclusion criteria and were included in the analysis. For the historical control, 629 BC signalled positive within the observed hours, of which 414 (65.8%) BC were included in the analysis (Suppl. Figure 2). Seven patients with identification by BF-FA-BCIP and four patients with conventional identification had two independent episodes of bacteraemia, resulting in a total of 789 unique patients.

Patient characteristics of the two groups were well balanced (Table 1). Overall, median age was 69 years, and 321 (40.1%) were female. Eighty-six of 386 cases (22.3%) and 80 of 414 controls (19.3%) had a qSOFA score of two or more at the time of BC collection. The main sources of BSI were urinary tract and intra-abdominal infection (19.2% vs. 22.0% and 16.8% vs. 15.7% for case and control groups, respectively). In total, 212 positive BC were considered as contamination (25.1% vs. 28.0%, respectively) (Table 1). Regarding the use of antimicrobials, patients received effective empiric therapy before BC positivity in 68.9% vs. 70.0% in the case and control group, respectively. Five of 386 cases (1.3%) and four of 414 controls (0.97%) did not receive effective treatment according to in vitro susceptibilities during hospitalisation.

**Table 1:** Summary of patient characteristics

	**Culture-based identification** (*n* = 414)	**BF-FA-BCIP** (*n* = 386)
Age, median [IQR^a^]	70 (56–79.8)	68 (58–78)
Female sex, n (%)	170 (41.1)	151 (39.1)
Charlson Comorbidity Index, median [IQR^a^]	2.0 [1.0–3.0]	2.0 [0.0–3.0]
qSOFA score at the time of BC collection, median [IQR^a^]	1.0 [0.0–1.0]	1.0 [0.0–1.0]
Patients with neutropenia, n (%)	49 (11.8)	45 (11.7)
Previous colonisation with MDRO^b^, n (%)	25 (6.0)	36 (9.3)
Allergy (Type I and IV), n (%)	52 (11.7)	45 (11.7)
Hospital Department, n (%)		
IMC/ICU	72 (17.4)	87 (22.5)
Medicine	248 (59.9)	207 (53.6)
Surgery	94 (22.7)	92 (23.8)
Source of bloodstream infection, n (%)		
Catheter-related	43 (10.4)	34 (8.8)
Urinary tract	91 (22.0)	74 (19.2)
Intra-abdominal	65 (15.7)	65 (16.8)
Respiratory tract	33 (8.0)	26 (6.7)
Skin/soft tissue	31 (7.5)	28 (7.3)
Intravascular	20 (4.8)	18 (4.7)
Contaminant	104 (25.1)	108 (28.0)

^a^IQR = interquartile range

^b^Multidrug-resistant microorganisms included: Methicillin-resistant *Staphylococcus aureus*, bacteria harbouring extended-spectrum beta-lactamases and Carbapenem-resistant gram-negative bacteria

Bacterial and fungal species identification was available after a median of 21.9 h (IQR 17.8, 25.7) by the BF-FA-BCIP and 44.3 h (IQR 39.6–50.6) hours by culture-based identification. More than half of positive BC grew Gram-positive bacteria (57.8%), while 39.5% were Gram-negative bacteria, 2.0% *Candida* species, and 13.6% grew multiple microorganisms (Suppl. Table 2). Two *Staphylococcus aureus* strains were methicillin-resistant, both belonging to the control group. No microorganisms with vancomycin-resistance or carbapenemases were identified.

### Clinical impact of the BF-FA-BCIP

**Fig. 1:**
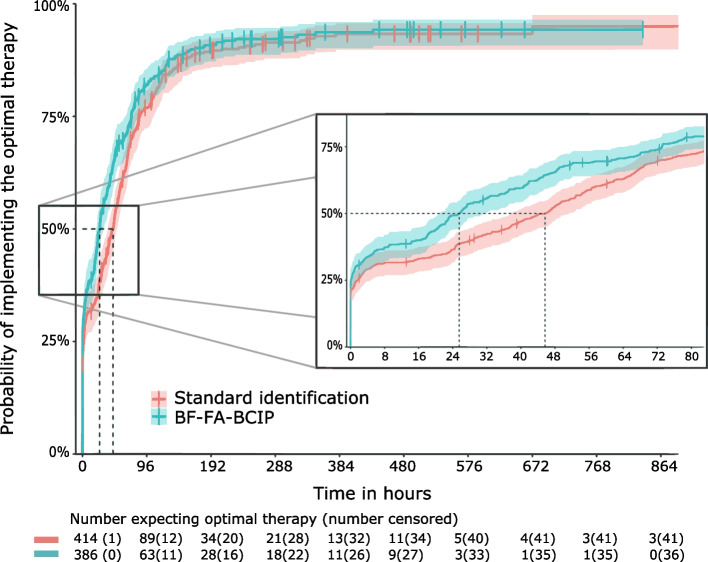
Cumulative incidence curve representing the probability of implementing the optimal therapy during hospitalisation. Time to optimal therapy was assessed using a cause-specific Cox proportional hazard model including the following covariates: hospital department, neutropenia, previous colonisation with MDRO and the CCI. Shaded ribbons represent the 95% confidence interval. Missing values due to death or discharge are censored to time of death or discharge (represented by vertical dashes)

Patients with identification by the BF-FA-BCIP received the OAT after a median of 25.5 h (95%CI 21.0—31.2) compared to 45.7 h (95%CI 37.7—51.2) in the control group (Fig. 1), resulting in a 21.5% higher chance to receive the OAT during hospitalisation (*p* = 0.010). We noted no effect on secondary outcome measures (Table 2). The implementation of the BF-FA-BCIP was not associated with a survival benefit. In both groups 36 patients died within hospital, and 46 of 379 case (12.1%) vs. 49 of 410 control (12.0%) patients died in the follow-up time of 30 days corresponding to an odds ratio of 1.1 for in-hospital and 1.0 for 30-day mortality. The time to effective antimicrobial therapy did not differ between the two groups and there was no significant difference in length of stay or ICU/IMC admission rates.

**Table 2:** Primary and secondary outcomes in relation to the identification method

	**Culture-based identification** **(*n* = 414)**	**BF-FA-BCIP (*n* = 386)**	**HR/OR [95% CI]**	***p*-value**
Primary outcome				
Time to optimal antimicrobial therapy in hours (median, (95%CI))	45.7 (37.7–51.4)	25.5 [21.0–36.4]	1.22 [1.05–1.39]	0.010
Secondary outcomes				
Time to effective antimicrobial therapy in hours (median, [IQR])	2.2 [0.1–23.5]	2.2 [0.0–19.0]	1.08 [0.94–1.25]	0.261
Length of stay after blood culture collection in days (median, [IQR])	10.1 [6.0–18.4]	11.6 [5.9–19.5]	0.97 [0.85–1.12]	0.696
30-day all-cause mortality^a^, n (%)	49 (12.0)	46 (12.1)	1.02 [0.66–1.56]	0.936
In-hospital mortality^a^, n (%)	36 (8.8)	36 (9.5)	1.09 [0.67–1.77]	0.726
(Re-)Admission to ICU/IMC after positive blood culture, n (%)	62 (15.0)	72 (18.7)	1.14 [0.74–1.75]	0.547

Model coefficients for time to effective therapy and length of stay are hazard ratios (HR), and odd ratios (OR) for mortality and (re-) admission to ICU/IMC. Values are presented with their 95% confidence interval (CI) or interquartile range (IQR). *P*-values are those associated with HR/OR

^a^For in-hospital and 30-day mortality, only unique patients were considered: *n* = 410 for culture-based identification, *n* = 379 for BF-FA-BCIP

In a subgroup analysis of hospital wards (medical, surgical, ICU/IMC), the BF-FA-BCIP was associated with decreased median times to OAT in all departments, however, the effect was significant only for the surgical wards. With the BF-FA-BCIP surgical patients (*n* = 186, 92 vs. 94 for case and control groups, respectively) received the OAT by a median of 34.3 h faster (21.1 h [95%CI 18.0–41.9] vs. 55.4 h [95%CI 46.1–68.7], respectively).

Primary and secondary outcomes remained similar through both study periods in a subgroup analysis of contaminated BC. Of patients with a contaminated BC, 93 of 108 (86.1%) with BF-FA-BCIP and 81 of 104 (77.9%) with standard culture-based identification did not receive any antimicrobial therapy. Use of intravenous vancomycin and daptomycin did not differ significantly between the two groups, with a median duration of 3 days [IQR 2–3] administered to 12 of 108 patients with identification by the BF-FA-BCIP and 3 days [IQR 1–4] administered to 13 of 104 patients with culture-based identification.

The BF-FA-BCIP was not associated with a significant effect on OAT in patients with neutropenia (*n* = 94, 45 vs. 49 for case and control groups, respectively). Median time to OAT did not differ significantly compared to immunocompetent patients.

### Diagnostic performance of the BF-FA-BCIP

The BF-FA-BCIP correctly identified all microorganisms in 97.4% of positive BC compared to subsequent culture-based identification (considering only on-panel microorganisms). 21 of 386 blood cultures grew microorganisms not covered by the BF-FA-BCIP resulting in a correct identification in 91.9% of all blood cultures.

At species level, sensitivity reached 98.5% (95%CI 95.6—99.7), and specificity reached 98.4% (95%CI 95.5—99.7). At genus level, sensitivity reached 99.2% (95%CI 97.7—99.8), and specificity reached 98.9% (95%CI 97.3—99.6). BF-FA-BCIP falsely identified microorganisms in four cases (1.0%) and failed to detect microorganisms in six cases (1.6%).

In four cases, the BF-FA-BCIP identified *Klebsiella pneumoniae*, whereas identification by MALDI-TOF MS resulted in *Klebsiella variicola*. *K. variicola* forms part of the *K. pneumoniae* complex and has only recently been described and added to the MALDI-TOF MS database [25]. Therefore, we considered these cases as true positive. Supplementary Table 3 shows a full report on false negative and false positive cases. In two cases, the BF-FA-BCIP identified a microorganism which did not grow in the subcultures and could therefore not be identified by culture-based identification.

## Discussion

In this retrospective single-centre study with a before-after design of 800 positive BC episodes, we report four major findings: (i) Rapid identification of microorganisms by the BF-FA-BCIP was associated with a median 20-h reduction of the time to optimal antimicrobial therapy compared to standard culture-based identification. (ii) This effect seemed to be most relevant for patients on surgical wards. (iii) The use of the BF-FA-BCIP had no impact on the time to effective therapy, length of stay, admission to ICU, and mortality. (iv) Sensitivity and specificity of the BF-FA-BCIP exceeded 98%, both for microorganisms identified on the genus as well as the species level.

The discussion on PCR-based identification techniques such as the BF-FA-BCIP has been ongoing for several years now. Next to some retrospective studies observing a faster initiation of OAT for example in ICU patients [8, 26], cancer patients [9] and paediatric patients [27], a three-armed prospective randomised trial was able to demonstrate a significantly earlier de-escalation of antimicrobial therapy for the implementation of the BF-FA-BCIP together with a real-time ASP [11]. Studies performed in the context of MDRO such as vancomycin-resistant enterococci have also been able to show earlier initiation of effective therapy, however without significant effects on clinical outcomes [10, 28]. A study on the cost-effectiveness of different molecular rapid diagnostic tests for diagnosis in suspected BSI showed evidence for lower health care costs in combination with an ASP [29]. A recent study focusing on the BF-FA-BCIP in *Escherichia coli* bacteraemia arrived at similar conclusions [30]. Still, the lack of evidence for a significant impact on hard clinical endpoints such as mortality leaves room for debate.

We believe that our study contributes to the accumulating data which supports the use of methods such as the BF-FA-BCIP and emphasises their value as a resource for greater sustainability in antimicrobial use [31, 32]. A 20-h faster availability of microorganism identification is relevant for clinical decision making and may be critical in severely ill patients. The impact of the BF-FA-BCIP on time to OAT demonstrates its utility as a part of antimicrobial stewardship strategies by reducing the exposure time to broad-spectrum antimicrobials. In our study, the time to effective therapy remained similar through the before and after study periods, possibly due to low rates of multi-drug resistance in our setting. The finding of a seemingly enhanced effect for patients hospitalised on surgical wards is interesting and may indicate that certain patient groups benefit more from rapid identification, however, the cause for this remains unclear.

In contrast to similar studies [7, 8, 33], we did not exclude contaminated BC or microorganisms not covered by the panel, representing a more real-world setting. At our hospital, the BF-FA-BCIP was introduced during hours of less staff availability, which was made possible by its minimal hands-on time. This demonstrates both a substantial advantage of the BF-FA-BCIP and a limitation of our study, as this may have led to an underestimation of the real effect. However, our results may be interesting to consider for centres with less human resources. In the present study, the BF-FA-BCIP misidentified four microorganisms and failed to identify six on-panel organisms leading to a delay in the initiation of optimal therapy in one case and to a delay in the initiation of effective therapy in one case. The misidentification of microorganisms is an important weakness which might compromise patient safety in rare cases. Another inherent limitation of the BF-FA-BCIP is the finite number of microorganisms that can be detected, however, in the present data set only 5% of bacteraemia episodes included off-panel microorganisms. Since the completion of our study, an updated version of the BF-FA-BCIP has been released, addressing this limitation as well as allowing identification of a greater variety of resistance genes.

Limitations to the generalisability of our results are the single-centre and retrospective design of our study. Rapid communication of microbiological results with written advice on antimicrobial therapy by infectious disease specialists may not be feasible for smaller institutions and implementing rapid diagnostic testing without an ASP seems to have a limited benefit [7, 11]. It remains unknown to which extent the written infectious disease consultation in the BF-FA-BCIP group may have influenced the primary outcome. Our study did not assess the cost-effectiveness which remains a restraining factor for its implementation, especially in smaller hospitals and low- and middle-income countries.

## Conclusions

In conclusion, patients with bacteraemia benefited from a median 20-h earlier initiation of the optimal antimicrobial therapy after the introduction of the BF-FA-BCIP at our centre. Our results demonstrate the potential of this method as a part of antimicrobial stewardship programmes even limited to certain hours per day. No impact on time to effective therapy, length of hospital stay and mortality was observed in our setting of low multi-drug resistance rates and a well-established ASP. Future studies could focus on identifying patient populations which benefit more from rapid microorganism identification and evaluate the BF-FA-BCIP in different settings such as high endemicity countries for MDRO and/or low- or middle-income countries.

### Supplementary Information

**Additional file 1: Supplementary Figure 1.** Hours during which the BF-FA-BCIP was performed. **Supplementary Figure 2.** Flow chart of positive BC assessed for eligibility, inclusion, and exclusion criteria. **Supplementary Table 1.** Definition of broadness of the antimicrobial therapy by microorganism. **Supplementary Table 2.** Identification of microorganisms in positive BC by standard culture-based identification in case and control groups. **Supplementary Table 3.** Patients with false positive (FP) or false negative (FN) microorganism identification by BF-FA-BCIP compared to subsequent standard culture-based identification. **Supplementary Material 1.1.** Detailed Description of Microbiological Analysis

## Data Availability

The data collected in the study is too voluminous or complex to be easily made available for public use. Interested parties may request specific parts of the data from the corresponding author AE.

## Abbreviations

BSI: Bloodstream infections
BC: Blood culture(s)
MDRO: Multi-drug resistant microorganism(s)
PCR: Polymerase chain reaction
BF-FA-BCIP: BioFire FilmArray blood culture identification panel 1
ASP: Antimicrobial stewardship programme
OAT: Optimal antimicrobial therapy
ICU: Intensive care unit
IMC: Intermediate care unit
qSOFA: Quick sequential organ failure assessment
CCI: Charlson comorbidity index
