# Supplementary material for: Identification of microorganisms by a rapid PCR panel from positive blood cultures leads to faster optimal antimicrobial therapy – a before-after study
Source: BMC Infect Dis. 2023 Oct 26;23:730. doi: 10.1186/s12879-023-08732-9 (PMC10601314; doi:10.1186/s12879-023-08732-9)
Supplement: Supplementary file 1 — Additional file 1: Supplementary Figure 1. Hours during which the BF-FA-BCIP was performed. Supplementary Figure 2. Flow chart of positive BC assessed for eligibility, inclusion, and exclusion criteria. Supplementary Table 1. Definition of broadness of the antimicrobial therapy by microorganism. Supplementary Table 2. Identification of microorganisms in positive BC by standard culture-based identification in case and control groups. Supplementary Table 3. Patients with false positive (FP) or false negative (FN) microorganism identification by BF-FA-BCIP compared to subsequent standard culture-based identification. Supplementary Material 1.1. Detailed Description of Microbiological Analysis [file 12879_2023_8732_MOESM1_ESM.pdf]

Supplementary Material

Supplementary Figure 1. Hours during which the BF-FA-BCIP was performed.

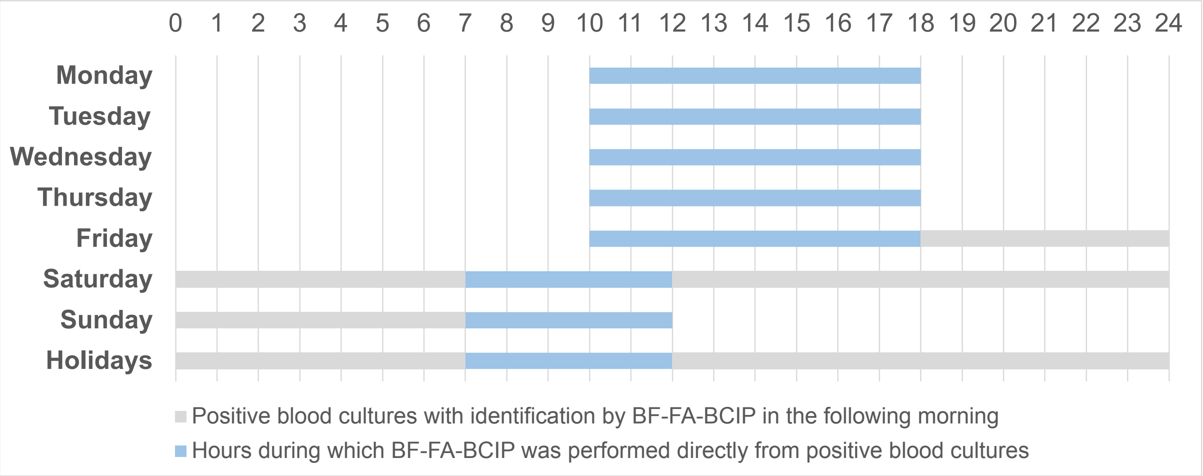

Supplementary Figure 2. Flow chart of positive BC assessed for eligibility, inclusion, and exclusion criteria. This flow diagram illustrates the positive BC assessed for eligibility, the resulting number of included BC; and the number and reasons for excluding BC based on our pre-determined criteria.

[#]TTP = time to positivity

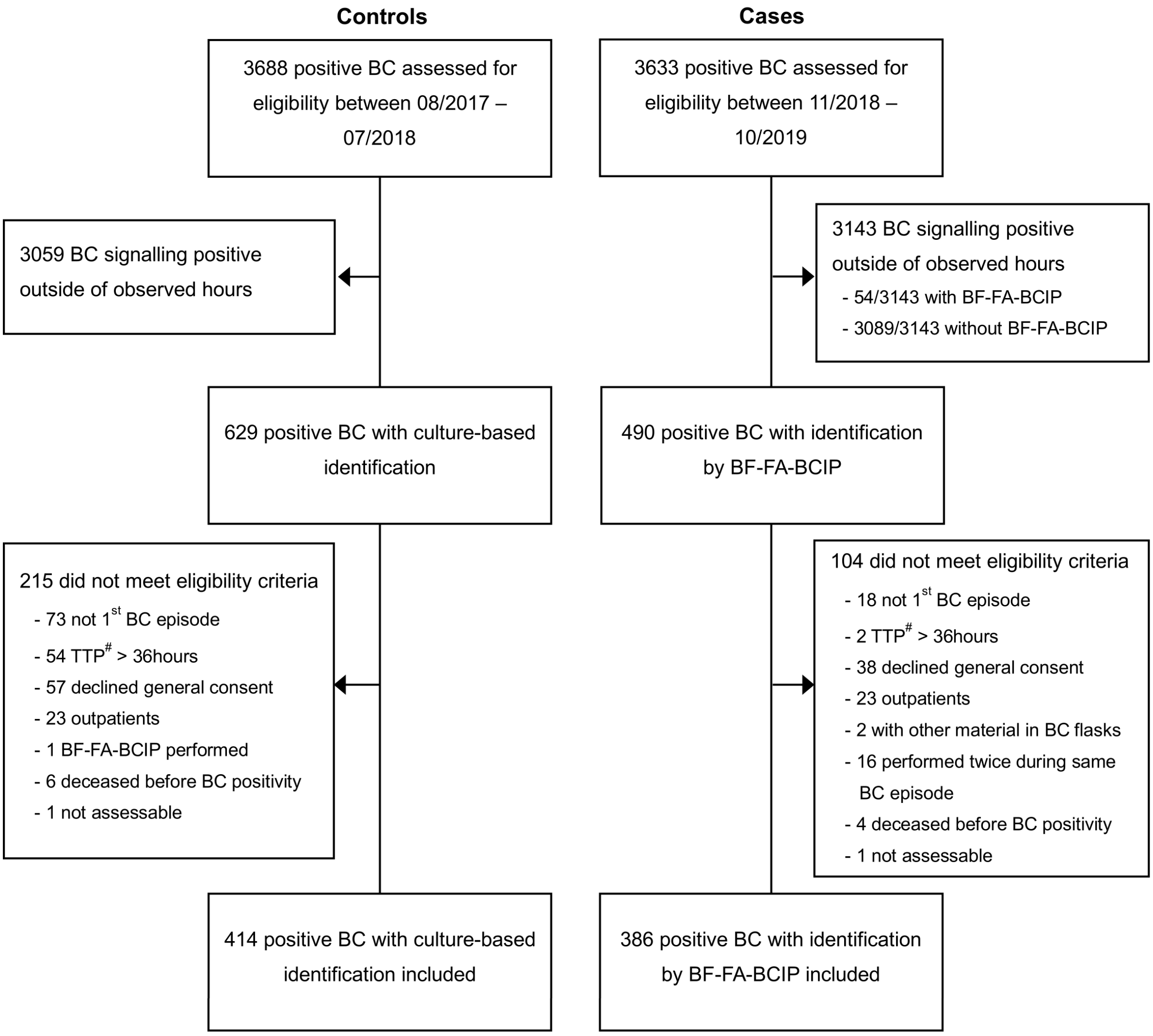

Supplementary Table 1. Definition of broadness of the antimicrobial therapy by microorganism.

Abbreviations: iv, intravenous administration; po, oral administration.

Identified Microorganism	Broad spectrum	Narrow spectrum	Optimal spectrum	Inappropriate
Staphylococcus spp.			Vancomycin (iv), Daptomycin (iv)	Amoxicillin/Clavulanic acid, Piperacillin/Tazobactam, Ertapenem, Imipenem, Meropenem
Staphylococcus aureus	Imipenem (iv), Cefepime (iv), Piperacillin/Tazobactam (iv)	Amoxicillin/Clavulanic acid (iv/po), Daptomycin (iv)	Flucloxacillin (iv), Penicillin (iv), Cefazolin (iv)	Ceftriaxone, Ertapenem, Meropenem, Vancomycin
Streptococcus spp.	Imipenem (iv), Piperacillin/Tazobactam (iv), Moxifloxacin (po), Levofloxacin (po)	Amoxicillin/Clavulanic acid (iv/po), Ceftriaxone (iv)	Penicillin (iv), Amoxicillin (po), Clindamycin (po)	Aztreonam
Streptococcus agalactiae	Imipenem (iv), Piperacillin/Tazobactam (iv), Moxifloxacin (po), Levofloxacin (po)	Amoxicillin/Clavulanic acid (iv/po), Ceftriaxone (iv)	Penicillin (iv), Amoxicillin (po), Clindamycin (po)	Aztreonam
Streptococcus pneumoniae	Imipenem (iv), Piperacillin/Tazobactam (iv), Moxifloxacin (po), Levofloxacin (po)	Amoxicillin/Clavulanic acid (iv/po), Ceftriaxone (iv)	Penicillin (iv), Amoxicillin (po), Clindamycin (po)	Aztreonam
Streptococcus pyogenes	Imipenem (iv), Piperacillin/Tazobactam (iv), Moxifloxacin (po), Levofloxacin (po)	Amoxicillin/Clavulanic acid (iv/po), Ceftriaxone (iv)	Penicillin (iv), Amoxicillin (po), Clindamycin (po)	Aztreonam
Enterococcus spp.	Imipenem (iv), Piperacillin/Tazobactam (iv)	Amoxicillin/Clavulanic acid (iv/po)	Amoxicillin (po), Vancomycin (iv), Daptomycin (iv), Linezolid (po)	Ceftriaxone, Ceftazidime, Cefepime
Listeria monocytogenes	Meropenem (iv), Imipenem (iv), +/- Aminoglycosides (iv)	Trimethoprim/Sulfamethoxazole (po)	Amoxicillin (po), Penicillin (iv/po)	Ceftriaxone, Ceftazidime, Cefepime, Flucloxacillin
Enterobacterales	Ertapenem (iv), Imipenem (iv), Meropenem (iv)	Piperacillin/Tazobactam (iv), Ceftazidime (iv), Cefepime (iv)	Amoxicillin/Clavulanic acid (iv/po), Ceftriaxone (iv), Ciprofloxacin (po)	Flucloxacillin, Penicillin

E. cloacae-Complex	Imipenem (iv), Meropenem (iv)		Cefepime (iv), Ciprofloxacin (po), Ertapenem (iv)	Penicillin, Amoxicillin, Amoxicillin/Clavulanic acid, Piperacillin/Tazobactam, Ceftriaxone, Ceftazidime
Escherichia coli	Ertapenem (iv), Imipenem (iv), Meropenem (iv)	Amoxicillin/Clavulanic acid (iv/po), Piperacillin/Tazobactam (iv), Ceftazidime (iv), Cefepime (iv)	Ceftriaxone (iv), Ciprofloxacin (po)	Flucloxacillin, Penicillin, Daptomycin, Vancomycin
Klebsiella oxytoca	Ertapenem (iv), Imipenem (iv), Meropenem (iv)	Amoxicillin/Clavulanic acid (iv/po), Piperacillin/Tazobactam (iv), Ceftazidime (iv), Cefepime (iv)	Ceftriaxone (iv), Ciprofloxacin (po)	Flucloxacillin, Penicillin, Daptomycin, Vancomycin
Klebsiella pneumoniae	Ertapenem (iv), Imipenem (iv), Meropenem (iv)	Amoxicillin/Clavulanic acid (iv/po), Piperacillin/Tazobactam (iv), Ceftazidime (iv), Cefepime (iv)	Ceftriaxone (iv), Ciprofloxacin (po)	Flucloxacillin, Penicillin, Daptomycin, Vancomycin
Proteus spp.	Ertapenem (iv), Imipenem (iv), Meropenem (iv)	Amoxicillin/Clavulanic acid (iv/po), Piperacillin/Tazobactam (iv), Ceftazidime (iv), Cefepime (iv)	Ceftriaxone (iv), Cefepime (iv), Ciprofloxacin (po)	Flucloxacillin, Penicillin
Serratia marcescens	Ertapenem (iv), Imipenem (iv), Meropenem (iv)		Cefepime (iv), Ciprofloxacin (po), Ertapenem (iv)	Penicillin, Amoxicillin, Amoxicillin/Clavulanic acid, Piperacillin/Tazobactam, Ceftriaxone, Ceftazidime
Pseudomonas aeruginosa	Imipenem (iv), Meropenem (iv)	Piperacillin/Tazobactam (iv), Cefepime (iv)	Ceftazidime (iv), Ciprofloxacin (po)	Penicillin, Amoxicillin, Amoxicillin/Clavulanic acid, Ceftriaxone
Haemophilus influenzae	Ertapenem (iv), Imipenem (iv), Meropenem (iv)	Amoxicillin/Clavulanic acid (iv/po), Piperacillin/Tazobactam (iv), Ceftazidime (iv), Cefepime (iv), Ceftriaxone (iv)	Amoxicillin (po), Ciprofloxacin (po)	Flucloxacillin
Acinetobacter baumannii	Ertapenem (iv), Imipenem (iv), Meropenem (iv)		Cefepime (iv), Ciprofloxacin (po), Ertapenem (iv)	Penicillin, Amoxicillin, Amoxicillin/Clavulanic acid, Piperacillin/Tazobactam, Ceftriaxone, Ceftazidime

Neisseria meningitidis	Ertapenem (iv), Imipenem (iv), Meropenem (iv), Piperacillin/Tazobactam (iv)	Amoxicillin/Clavulanic acid (iv/po), Ceftriaxone (iv)	Penicillin (iv)	Flucloxacillin
Candida albicans	Amphotericin B (iv), Caspofungin (iv), Anidulafungin (iv)		Fluconazole (po)	
Candida glabrata	Amphotericin B (iv)		Caspofungin (iv), Anidulafungin (iv)	Fluconazole
Candida krusei	Amphotericin B (iv)	Caspofungin (iv), Anidulafungin (iv)	Voriconazole (po), Posaconazole (po), Isavuconazole (po)	Fluconazole
Candida parapsilosis	Amphotericin B (iv), Caspofungin (iv), Anidulafungin (iv)		Fluconazole (po)	
Candida tropicalis	Amphotericin B (iv), Caspofungin (iv), Anidulafungin (iv)		Fluconazole (po)	
mecA		Linezolid (iv/po)	Vancomycin (iv), Daptomycin (iv)	Amoxicillin/Clavulanic acid, Piperacillin/Tazobactam, Ertapenem, Imipenem, Meropenem
vanA/B		Linezolid (iv/po), Tigecycline (iv)	Daptomycin (iv), Linezolid (iv/po)	Vancomycin, Amoxicillin, Imipenem
KPC		Ceftazidime/Avibactam (iv), Colistin (iv), Aztreonam/Avibactam (iv), or as combined therapy		Penicillin, Amoxicillin, Amoxicillin/Clavulanic acid, Piperacillin/Tazobactam, Ceftriaxone, Ceftazidime, Cefepime, Meropenem, Imipenem, Ertapenem, Ceftarolin, Ceftobiprole, Ceftolozan/Tazobactam

Supplementary Table 2. Identification of microorganisms in positive BC by standard culture-based identification in case and control groups. Listed according to on-panel microorganisms of the BF-FA-BCIP. Please note that numbers do not sum up due to polymicrobial growth in BC (11.1% vs. 12.1% for case and control groups, respectively).

Abbreviations: spp, species.

* Includes: *Bacillus* spp., *Micrococcus* spp., *Corynebacterium* spp., *Lactobacillus* spp., *Lactococcus* spp., *Actinotignum schaalii*, *Ruminococcus gnavus*, *Cutibacterium acnes*, *Finegoldia magna*, *Granulicatella adiacens*

# Includes: *Campylobacter* spp., *Bacteroides* spp., *Acinetobacter* spp. (not *A. baumanii*), *Veillonella* spp., *Prevotella* spp., *Capnocytophaga sputigena*, *Stenotrophomonas maltophilia*, *Neisseria subflava*.

	**Controls** (n= 414)	**Cases** (n=386)
**Gram-positive microorganisms, n (%)**	**263 (63.5)**	**271 (70.2)**
Staphylococcus aureus	39	37
Coagulase-negative *Staphylococci*	131	135
Streptococcus spp. (not *S. pneumoniae, S. pyogenes* or *S. agalactiae*)	39	37
Streptococcus pneumoniae	13	15
Streptococcus pyogenes	4	3
Streptococcus agalactiae	3	3
Enterococcus spp.	19	33
Listeria monocytogenes	0	0
Other not on-panel microorganisms*	15	8
**Gram-negative microorganisms, n (%)**	**195 (47.1)**	**171 (44.3)**
Escherichia coli	112	87
Klebsiella pneumoniae	25	17
Klebsiella oxytoca	3	6
Enterobacter spp.	6	10
Pseudomonas aeruginosa	21	14

Haemophilus influenzae	1	1
Serratia marcescens	4	5
Acinetobacter baumannii	0	0
Neisseria meningitidis	2	0
Proteus spp.	4	1
Other *Enterobacterales*	8	17
Other not on-panel microorganisms #	9	13
**Yeast, n (%)**	**10 (2.4)**	**6 (1.6)**
Candida albicans	5	2
Candida glabrata	4	1
Candida krusei	0	0
Candida parapsilosis	0	1
Candida tropicalis	1	0
other *Candida* spp.	0	2

Supplementary Table 3. Patients with false positive (FP) or false negative (FN) microorganism identification by BF-FA-BCIP compared to subsequent standard culture-based identification.

Patient ID	Culture-based Identification	BF-FA-BCIP	FP (species level)	FP (genus level)	FN (species level)	FN (genus level)
BIOFIRE-USB-12910	*Staphylococcus epidermidis*	*Staphylococcus* spp, *Enterobacteriaceae*	No	Yes	No	No
BIOFIRE-USB-12270	*Enterococcus faecium*	negative	No	No	No	Yes
BIOFIRE-USB-13460	*Morganella morganii*	negative	No	No	No	Yes
BIOFIRE-USB-10190	*Bacteroides fragilis*	*Enterobacteriaceae, Proteus*	Yes	Yes	No	No
BIOFIRE-USB-11550	*Escherichia coli*	negative	No	No	Yes	Yes
BIOFIRE-USB-10100	*Klebsiella oxytoca*	*Enterobacteriaceae*	No	No	Yes	No
BIOFIRE-USB-10930	*Klebsiella aerogenes*	*Enterobacteriaceae, Klebsiella pneumoniae*	Yes	No	No	No
BIOFIRE-USB-13140	*Klebsiella aerogenes*	*Enterobacteriaceae, Klebsiella pneumoniae*	Yes	No	No	No
BIOFIRE-USB-13210	*Serratia marcescens, Enterobacter cloacae, Klebsiella* species	*Enterobacteriaceae, Serratia marcescens*	No	No	Yes	No
BIOFIRE-USB-14270	*Staphylococcus epidermidis, Streptococcus mitis, Enterobacter cloacae, Enterococcus avium, Pseudomonas aeruginosa*	*Streptococcus* spp, *Enterococcus* spp, *Enterobacteriaceae, Enterobacter cloacae, Pseudomonas aeruginosa*	No	No	No	Yes

Supplementary Material 1.1. Detailed Description of Microbiological Analysis

In positive BC with gram-negative rods, species identification is based on biochemical profiling (VITEK2 GN card, bioMérieux). 5mL of BC material is transferred to a Vacutainer serum tube (BD, Allschwil, Switzerland) and centrifuged at 2000g for 10min. The cloudy supernatant represents bacterial cells and is transferred to NaCl 0.45% solution to make a 0.5-0.63 Mc Farland suspension. This suspension is used in the VITEK2 GN card for biochemical identification and susceptibility testing (AST-N242 card).

For overnight subculture, the following plates were used (depending on the results of the direct microscopy): gram-negative rods are subcultured on 5% sheep blood agar (BA, bioMérieux), chromogenic ID agar (CHROM, bioMérieux), Brucella Agar (BRU, BD), Schaedler 5% sheep blood agar with Kanamycin and Vancomycin (KV, BD). In case of morphotype *Haemophilus* we used BA, chocolate agar PolyViteX agar (PVX, bioMérieux), BRU and KV. In case of morphotype *Campylobacter*, we used BRU and BA incubated at 36° and 42°C under microaerophilic conditions. Gram-negative diplococci are subcultured with BA, KB, and BRU. Gram-positive cocci were incubated with BA and BRU. Gram-positive rods were incubated at BA and BRU. Yeast was incubated on BA, Sabouraud without antibiotics (SAB, BD), Candida Chrom Agar (CAN, bioMérieux), and BRU. Single colonies were identified by MALDI-TOF MS using the Microflex System (Bruker, Bremen, Germany) according to the manufacturer's instructions. For susceptibility testing of gram-positive bacteria, a 0.5-0.63 Mc Farland suspension is made as described above and used in the VITEK2 AST-P586 and AST-P636 cards.
